# HIV-Associated Tuberculosis in Children and Adolescents: Evolving Epidemiology, Screening, Prevention and Management Strategies

**DOI:** 10.3390/pathogens11010033

**Published:** 2021-12-29

**Authors:** Alexander W. Kay, Helena Rabie, Elizabeth Maleche-Obimbo, Moorine Penninah Sekadde, Mark F. Cotton, Anna M. Mandalakas

**Affiliations:** 1 Global Tuberculosis Program, Department of Pediatrics, Baylor College of Medicine and Texas Chidlren’s Hospital, Houston, TX 77030, USA; anna.mandalakas@bcm.edu; 2 Department of Pediatrics and Child Health and FAMCRU, Stellenbosch University and Tygerberg Hospital, Cape Town 7505, South Africa; hrabie@sun.ac.za; 3 Department of Pediatrics and Child Health, University of Nairobi, Nairobi 00202, Kenya; lisaobimbo@gmail.com; 4 National Tuberculosis and Leprosy Programme (NTLP), Kampala 7272, Uganda; moorine.sekadde@gmail.com; 5 Children’s Infectious Diseases Clinical Research Unit, Department of Paediatrics and Child Health, Faculty of Medicine and Health Sciences, Stellenbosch University, Cape Town 7505, South Africa; mcot@sun.ac.za

**Keywords:** tuberculosis, HIV, child, adolescent

## Abstract

Children and adolescents living with HIV continue to be impacted disproportionately by tuberculosis as compared to peers without HIV. HIV can impact TB screening and diagnosis by altering screening and diagnostic test performance and can complicate prevention and treatment strategies due to drug–drug interactions. Post-tuberculosis lung disease is an underappreciated phenomenon in children and adolescents, but is more commonly observed in children and adolescents with HIV-associated tuberculosis. This review presents new data related to HIV-associated TB in children and adolescents. Data on the epidemiology of HIV-associated TB suggests that an elevated risk of TB in children and adolescents with HIV persists even with broad implementation of ART. Recent guidance also indicates the need for new screening strategies for HIV-associated TB. There have been major advances in the availability of new antiretroviral medications and also TB prevention options for children, but these advances have come with additional questions surrounding drug–drug interactions and dosing in younger age groups. Finally, we review new approaches to manage post-TB lung disease in children living with HIV. Collectively, we present data on the rapidly evolving field of HIV-associated child tuberculosis. This evolution offers new management opportunities for children and adolescents living with HIV while also generating new questions for additional research.

## 1. Introduction

This review focuses on recent evidence regarding tuberculosis (TB) incidence, prevention, diagnosis and management in children and adolescents living with human immunodeficiency virus infection (CALHIV). In light of decreasing TB case notifications globally and increasing TB mortality associated with the SARS-CoV-2 (COVID-19) pandemic [1], it is a critical time to renew the commitment to improving TB management in highly vulnerable populations such as CALHIV.

## 2. Emerging Epidemiology on TB Risk and Outcomes in High-HIV/TB Burden Settings

The epidemiology of HIV-associated TB in children and adolescents continues to evolve along with advances in HIV care guidelines [2]. Despite the widespread introduction of antiretroviral therapy (ART), CALHIV remains at high risk for HIV associated TB even in cohorts with normal CD4 counts and viral suppression. This ongoing risk was demonstrated in two South African studies. The first demonstrated that adolescents with perinatally acquired HIV had a TB incidence of 2.2/100 person years (PY) (95% confidence interval (CI) 1.6 to 3.1) compared to adolescents without HIV with a TB incidence of 0.3/100 PY (95% CI 0.04 to 2.2); these observations suggest an incidence rate ratio (IRR) of 7.4 (95% CI 1.01 to 53.6) [3]. Although the cohort’s median CD4 count was 713 cells/m^3^ and 76% had a viral load of <40 copies/mL, CALHIV with a CD4 count <500 cells/m^3^ and a viral load above 1000 copies/mL had an increased risk of TB disease.

Evidence from other African settings also demonstrated the persistent risk of HIV-associated TB despite ART (Table 1). In a cohort of CALHIV in Ethiopia, ART only reduced the hazard ratio of TB by 36% [4]. The TB incidence rate was 7.7 per 100 person years (95% CI, 6.3–9.2) in CALHIV on ART, similar to 8.2 per 100 person years (95% CI 6.8–9.8) in ART naïve CALHIV. A similar increased risk was observed in a cohort of infants in Kenya were observed in infants with HIV (adjusted hazards ratio (aHR) 4.71 (2.13–10.4) [5]. In a cohort of CALHIV observed in 6 sub-Saharan African countries, increases in ART were associated with declines in TB period prevalence but even with ART coverage nearing 100%, CALHIV still developed TB at approximately 2 per 100 person years [6]. 

This evidence aligns with data from a recent systematic review indicating that HIV remains a strong risk factor for incident and prevalent TB in children following close TB exposure [7]. Notably, the evidence demonstrates that most cases of TB occurred in the 3 months following exposure. Hence, there is a clear need to develop systems that rapidly identify CALHIV and those who can benefit from tuberculosis preventive therapy (TPT). As multi-month prescribing for CALHIV is implemented more broadly, programs will need to develop thoughtful screening strategies to identify TB exposure and TB disease early even if children are not presenting on a monthly basis to obtain ARVs. 

CALHIV also continue to have worse treatment outcomes than peers without HIV. A South African study compared programmatic TB treatment outcomes in 729,463 children and adolescents with and without HIV and treated for TB between 2004 and 2016 [8]. Although the study demonstrated a decline in the overall case fatality ratio over time, HIV remained a risk factor for mortality (adjusted hazard ratio (aHR) = 5.11 (95% confidence interval 4.71–5.55) even after the initiation of ART. Another study evaluating program data in Kenya (n = 24,214) demonstrated a similar aHR of 4.84 (95% confidence interval 3.59–6.91) among CALHIV not on ART and aHR of 3.69 (95% confidence interval 3.14–4.35) in CALHIV on ART. 

Collectively, this data demonstrates that while ART effectively reduces TB risk and improves outcomes among CALHIV in sub-Saharan Africa, the risk remains highly elevated compared to populations without HIV. As a whole, these findings may reflect the persistently high force of TB infection in these settings, as data from low burden settings suggests a more significant decline in HIV-associated TB attributable to ART uptake [9]. This persistent risk of incident TB coupled with an elevated risk of mortality from HIV-associated TB indicates the ongoing need for novel TB screening, diagnostic and preventive strategies for CALHIV. This ongoing risk in CALHIV on ART is unfortunately exacerbated by the high proportion, nearly 50%, of children still not accessing ART globally [10].

There are additional intersections between TB and COVID-19 to be considered. Adult data suggests that TB and HIV are both associated with an increased risk of mortality from COVID-19. Individuals with TB have a 2.7-fold increased risk of COVID-19 mortality (aHR) 2.7, 95% CI 1.8 to 4.0) [11]. Similarly, PLHIV have a 2.1-fold increased risk of COVID-19 mortality (aHR 2.1, 95% CI 1.7 to 2.7). Data from a single center in South Africa reported that only 2 of 62 children hospitalized in the first 6 months of the pandemic had HIV, but surveillance data also from South Africa reports that 50 of 565 (8.8%) adolescents and children who died from COVID-19 also had HIV [12]. This evidence suggests that CALHIV may be over-represented among COVID-19-related deaths in these age groups. 

Global TB notification rates declined from 7.2 to 5.9 million in 2020 [1]. These declines in part reflect COVID-19 induced disruptions in TB health services. Nevertheless, it is unclear if masking and social distancing alter adult and children’s risk of *Mycobacterium tuberculosis* infection and subsequent disease. Of note, epidemiological data from South Africa shows that pediatric TB diagnosis increases soon after the peak of the epidemiological curve of influenza; it remains to be seen whether an increase may be observed following the COVID-19 pandemic as well [13]. In addition, patients may seek care later. Care at the clinic may be fragmented and diagnostic procedures be delayed for fear of COVID-19, and health care resources may have shifted to COVID-19. Early program data from South African hospitals demonstrate a reduction in TB case notifications and overall pediatric admissions suggesting a change in health seeking behavior and a weakening of existing TB health systems for TB diagnosis [14]. The long-term impact of COVID-19 on TB and HIV-associated TB in children must be watched closely. 

## 3. TB Screening

The probability of TB progression and poor outcomes is higher in CALHIV than their peers without HIV, partly due to diagnostic delays and non-specific presentation. One recommended vital strategy for early TB case detection is systematic TB screening, especially for high-risk groups such as CALHIV. However, evidence to inform the design of pediatric TB screening strategies is limited. As a result, the implementation of TB screening in children remains a challenge and has contributed to the significant gap in TB case detection. 

Advances in TB screening approaches for children generally lag behind that of adults due to limited evidence, often leading to the adaptation of evidence-based adult recommendations despite differences in disease characteristics. More recently, the World Health Organization recommended that systematic screening for TB disease be conducted using a symptom screen including any one of current cough, fever, poor weight gain, or close contact with a TB patient among children <10 years who are living with HIV at each care encounter. Any child living with HIV with a positive symptom screen should undergo further diagnostic evaluation [16], but programmatic data from Kenya suggests attendance at pediatric-focused HIV clinics may be associated with a lower likelihood of appropriate diagnostic follow up [17]. Additionally, as this approach misses up to 40% of children with HIV-associated TB, research on new strategies for TB screening in this high-risk population is urgently needed [18]. 

**Figure 1:**
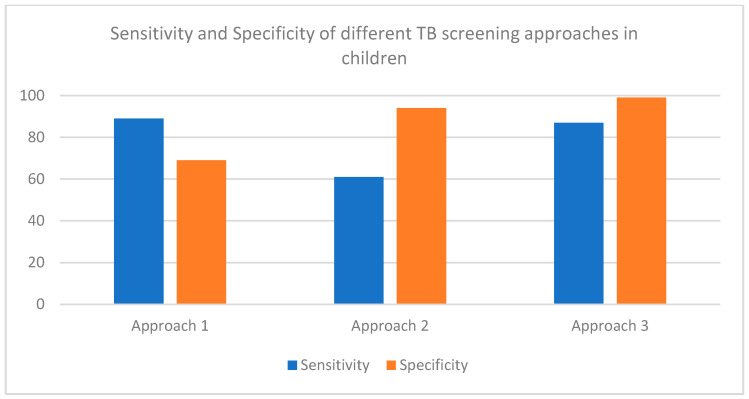
TB screening approaches in children <10 years. The performance of screening strategies was compared via evidence identified through a systematic review of the literature [19]. Approach 1: One or more of cough, fever, or poor weight gain in tuberculosis contacts (evidence derived from 4 studies with tuberculosis prevalence ranging from 2% to 13%). Approach 2: One or more of cough, fever, poor weight gain, or tuberculosis close contact (World Health Organization four-symptom screen) in children living with HIV, outpatient (evidence derived from 2 studies with tuberculosis prevalence ranging from 3% to 8%). Approach 3: CXR with any abnormality in tuberculosis contacts (evidence derived from 8 studies with tuberculosis prevalence ranging from 2% to 25%).

Advancement in HIV care, including the provision of differentiated service delivery approaches, has created new opportunities to effectively integrate TB screening within routine HIV services and ensure uninterrupted care. Similarly, systematic screening for TB disease should be conducted using a symptom screen including any one of cough, fever or poor weight gain; or chest radiography; or both for children <15 years with TB exposure (Figure 1). The yield of symptom screening is higher among close contacts of TB patients than other risk groups. If available, CXR may be used as a TB screening tool for children <15 years with TB exposure [19]. Direct comparisons of these strategies in CALHIV were not performed. The use of C-reactive protein in TB screening is currently limited to adolescents and adults living with HIV [16]. Commercially available tests of TB infection (TST and IGRA) cannot differentiate between TB infection and disease, may be affected by conditions unrelated to TB infection, and are often inaccessible in TB high-burden settings. Hence, tests of infection have a limited role in TB screening in high-burden settings, particularly among CALHIV.

## 4. Prevention Strategies in the Context of Anti-Retroviral Therapy

The elevated TB risk among CALHIV despite anti-retroviral therapy (ART) indicates the ongoing need for effective and widespread implementation of TB preventive treatment (TPT) in this population. Data on drug–drug interactions between rifamycins and dolutegravir is urgently needed so that CALHIV may also benefit from the increasing availability of shorter TPT treatment regimens. Recent data suggests that 1 month of isoniazid and rifapentine is safe and feasible in children and adolescents two years and above [20]. This data, combined with efficacy data in adults living with HIV suggests that this very short TPT regimen, is appropriate for CALHIV [21]. However, there is limited evidence to inform whether adjustments in dolutegravir dosing are needed in children receiving daily or weekly rifapentine for TPT.

While more data is needed to optimize TPT delivery for CALHIV, existing data on TPT uptake and completion is encouraging. While TPT uptake is more robust in adults than children, rates of TPT adherence and completion have been quite good when evaluated within observational cohorts of CALHIV. Evidence demonstrates that differentiated service delivery models, TPT education, and seamless integration with HIV care are important predictors of TPT completion [22,23,24]. Given persistently high rates of TB disease among CALHIV, TPT scale up is essential and will benefit from shorter TPT regimens, child-friendly formulations, and patient-centered TPT administration integrated within ART to minimize increased burden on patients and health systems.

## 5. Diagnostic Strategies for HIV-Associated TB

The TB case detection gap in CALHIV remains problematic and is exacerbated by the COVID-19 pandemic [1]. However, new developments in diagnostics may reduce this gap. Building on years of work supporting the value of stool and nasopharyngeal aspirates for child TB diagnosis, the WHO now endorses both specimen types for TB diagnosis in CALHIV [25,26]. Stool specimens and nasopharyngeal aspirates have similar sensitivity to respiratory specimens when combined or only slightly lower than respiratory specimens as stand-alone tests. However, as these specimens are easier for nurses to collect and require less equipment, more children, including CALHIV, should be evaluated for TB. Additional implementation science work is urgently needed to evaluate the impact of collecting these non-traditional specimens in CALHIV. New diagnostic approaches will certainly be needed as only a small minority of CALHIV on TB treatment have a confirmation by Xpert [26]. Additionally, there is emerging data for urine as a useful specimen for TB diagnosis with the new SILVAMP-LAM point-of-care assay. 

One report from a South African pediatric cohort demonstrated a 24% increase in sensitivity in CALHIV (60%, 95%CI 40.7–76.6 for SILVAMP-LAM vs. 36%, 95%CI, 20.2–55.5 for Alere-LAM) against a microbiologic reference standard [27]. SILVAMP-LAM performed equally well in stored urine specimens from 4 African pediatric TB clinics. Sensitivity was 64.9%, 95% CI 43.7–85.2; positive in 40 of 63 confirmed samples versus 30.7% (95% CI 8.6–61.6; 19 positive of 63 confirmed samples) for Alere-LAM [28]. The specificity was similar for both tests in each study; however, additional data using prospectively collected specimens is needed to confirm these promising results. 

While new bacteriological tests are important, they are unlikely to identify all cases of paucibacillary TB in CALHIV. Fortunately, emerging approaches to support clinical diagnosis have great promise. Leveraging data from a diagnostic study of 438 CALHIV, a treatment-decision score was developed based on detailed symptom assessments and radiographic data, including CXR and ultrasound; the resultant score had an AUC of 0.861 with a sensitivity of 88.6% and a specificity of 61.2% compared to a composite tuberculosis diagnosis using the NIH consensus definitions [29]. Although CXR was an important component of the algorithm, many high-risk children with exposure to TB and prolonged TB symptoms, can be diagnosed correctly with this algorithm without a CXR. Further, this study also highlighted poor agreement between local radiologists, pediatric pulmonologists and pediatric radiologists and limited overall diagnostic accuracy (55.3%) in CALHIV [30]. Hence, while CXR may play a role in TB screening or diagnosis for CALHIV, new strategies, perhaps including computer aided detection, are needed to increase the accuracy of this important tool. Preliminary data on complementary approaches using monocyte-to-lymphocyte ratios as a diagnostic aid also merits additional exploration [31].

## 6. Ongoing Challenges Navigating Co-Treatment and Drug-Drug Interactions in Children with HIV-Associated TB

When indicated, most CALHIV will receive TB prevention and treatment regimens in accordance with recommendations of the local tuberculosis programs. As these regimens usually comprise short-course therapy with fixed dose combinations, most children will receive rifampicin with isoniazid, pyrazinamide and ethambutol for treatment and isoniazid (possibly with rifapentine) for prevention. Rifampicin, isoniazid, pyrazinamide and ethambutol dosages will not require adjustment, although some data suggest that HIV infection alone results in lower tuberculosis drug levels [32]. There is currently no indication that different dosing is required for children with HIV-associated TB compared to child TB in general.

Rifamycins are key drugs for both tuberculosis treatment and prevention. All rifamycins affect the pharmacokinetic parameters of most antiretroviral drugs through their strong induction of cytochrome (CYP) P450, UDP- glucuronosyltransferase (UGT) and P-glycoprotein (P-gp). This induction reduces antiretroviral exposure and is an important cause of virological failure for CALHIV. Therefore, establishing treatment regimens that allow for co-administration of the most highly effective ART regimens with rifamycin based TB treatment is critical for children [32]. In Table 2, the dose adjustment suggested for children requiring co-treatment with rifampicin is summarized. This altered dosing should be continued for 7–14 days after stopping the rifampicin to allow the rifampicin-associated induction of CYP 450 to end. 

There are fewer data regarding drug interactions between rifabutin and antiretrovirals; however, the bidirectional reactions require careful consideration. Emerging data suggest that in children 3 years of age and above, rifabutin may be dosed at 2.5 mg/kg/day if given with lopinavir/ritonavir [33]. Although neutropenia appears to be less common than previously reported [34], regular monitoring remains advisable. There are no pediatric data to inform rifabutin dosing when given with dolutegravir, efavirenz, darunavir or atazanavir; hence, rifabutin is primarily used to treat HIV-associated TB in children who require lopinavir/ritonavir. There is no pediatric data to inform rifapentine dosing when given with antiretroviral drugs. Fortunately, studies are planned that will consider both weekly and daily rifapentine dosing with isoniazid in children who need TB preventative therapy or treatment. In children receiving weekly rifapentine with dolutegravir, it is unlikely that dose adjustment is needed based on adult data.

Critically, there is increasing data to suggest that dolutegravir can be administered with rifampicin in CALHIV. Pharmacokinetic data suggest that 50 mg of dolutegravir administered twice daily can be given with rifampicin in children over 6 years of age and 20 kg [35]. Further, the WHO has extrapolated the recommendation for twice daily dosing of standard dose dolutegravir co-administered with rifampicin for CALHIV of all ages [2]. While the evidence to inform the guidelines is limited, this will allow (for the first time in many high-burden settings) the youngest infants and children to receive the most effective ART regimen available when on rifampicin-based TB treatment. 

Although isoniazid inhibits some sub-classes of CYP P450, this inhibition is insufficient to counteract the drug–drug interactions associated with tuberculosis and HIV co-treatment in most children. Nevertheless, joint administration of isoniazid and ART may occasionally contribute to toxicity. In general, treatment for tuberculosis and HIV can have similar adverse effects and co-treatment poses a significant pill burden that mandates adherence support and clinical review for adverse drug reactions. 

When co-treating children with TB and HIV, time on therapy, clinical and immune staging and viral suppression should be reviewed. It is important to identify and manage malnutrition, other infections and HIV complications that often co-exist in the same child. Drug interactions should be reviewed not only between anti-tuberculosis medications and antiretrovirals but for all medications that may interfere with drug levels, for instance iron supplementation in children on dolutegravir. In Box 1, we highlight some key considerations. There is no recommendation to prolong anti-tuberculosis treatment in CALHIV, but all require careful monitoring and recovery should be documented.

Box 1 **Considerations for choosing a co-treatment regimen.**

**For anti-tuberculosis therapy, consider the following:**

Does the child require TB treatment or prevention?
What is the confirmed or most likely tuberculosis drug susceptibility pattern?
Are there additional considerations for choosing drugs? For instance, meningitis requires that medication crosses the blood–brain barrier and potentially higher doses.
Are there conditions that require consideration, for instance, severe anemia or electrolyte disorders that may complicate the use of some second line anti-tuberculosis drugs for instance linezolid and severe anemia.
If you are treating meningitis and the child is not already on antiretrovirals then delay the initiation of antiretroviral therapy for 4 weeks; otherwise, start within 2 weeks [36]. In hospitalized children initiate TB therapy and if possible antiretroviral therapy prior to discharge. Ensure counselling is performed and that and they are referred to the appropriate outpatient service.

**To choose the antiretroviral therapy:**

Can the available antiretrovirals be adjusted to maximize viral suppression while treating or preventing tuberculosis?
Are there any drug–drug interactions that should be considered? Remember to consider co-morbidities that the child may have and the therapy needed for those, for example, anticonvulsants.
Will the selected TB and HIV regimen require additional monitoring needs? If yes, does the child have access to these monitoring tools?
How will adherence be supported?

**For the child already on antiretroviral therapy:**

How long has the child been on therapy? Children who recently initiated antiretroviral therapy may develop IRIS and possibly need steroid therapy.
Is the child adherent, immune reconstituted and with a suppressed viral load? Children with severe immune suppression who are failing antiretroviral therapy are more likely to progress from TB infection to disease. Hence, switching antiretroviral therapy may be needed.
For children failing antiretroviral therapy—what is the most appropriate time to switch the antiretroviral regimen and which regimen will be required? Will the preferred antiretroviral regimen be compatible with the anti-tuberculosis regimen?

As shorter TPT regimens have become available, there have also been advances in shortening the treatment for TB disease in children. A phase 3 randomized clinical trial in 1204 children with minimal pulmonary disease, including 127 CALHIV, suggest that shorter courses of four months (2HRZE/2 HR) are as effective as 6 months of treatment [37]. In adolescents 12 years or older living with HIV, and with CD4 counts ≥100 cells/mL^3^, treatment shortening with two months of isoniazid, rifapentine, moxifloxacin and pyrazinamide, followed by an additional two months of isoniazid, rifapentine moxifloxacin was non-inferior to a standard 6 months regimen [38]. Data on the implementation of both of these regimens is needed, but for the first time in over 50 years data supports treating TB disease for less than 6 months, inclusive of CALHIV.

Over the past decade, considerable effort has been exerted to integrate TB and HIV care and treatment for people living with HIV. Emerging evidence suggests that the integration of care improve TB and HIV outcomes for CALHIV [6]. As highlighted in this review, new options for the prevention and treatment of TB are now available and more are expected in the coming years. The effective integration of TB and HIV care will help to ensure that these new therapeutic options reach all CALHIV. 

## 7. Improving Care after Treatment for TB Disease

Effective delivery of ART has improved survival from TB among CALHIV. However, emerging evidence suggests that a significant proportion of TB survivors have residual post-TB lung disease (PTLD) following more severe forms of pulmonary or extrapulmonary TB. Accurate data regarding children surviving HIV-associated TB disease are not readily available. It is estimated that ~15% of the 1.72 million CALHIV <15 years living have had prior TB, forming a large at risk group for PTLD [10,39]. 

In recent adolescent observational studies from sub-Saharan Africa, adolescents surviving HIV-associated TB had 3.15 fold increased odds of abnormal lung function, including reduced air flow and lung volumes compared to those without prior TB history [40,41]. Evidence on TB sequelae in younger children is lacking, despite the fact that young children comprise the highest numbers of severe cases of TB, and are thereby at high risk of long-term PTLD.

The pathophysiology of chronic PTLD is thought to be multifactorial largely due to chronic inflammation triggered by prolonged *M. tuberculosis* infection of affected tissues, and slow resolution of infection in the immune-compromised HIV+ child even after starting anti-TB treatment [42,43]. HIV infection itself causes chronic systemic inflammation and immune dysregulation which causes end-organ damage, including the lung [44,45]. There is resultant irreversible injury to affected tissues. Pathologic changes include bronchial wall fibrotic changes, with obstruction, plugging and/or dilatation, causing chronic obstructive airway disease. In addition, lung matrix injury may manifest with fibrosis in segments of lung parenchyma with volume loss. Pleural damage may manifest as pleural thickening, with resultant restrictive lung disease [46].

Clinical presentation depends on the site and extent of injured tissue, and may range from subclinical and asymptomatic to severely symptomatic disease with significant lung function impairment. The commonest clinical presentation for more severe forms of PTLD in children is persistent respiratory symptoms such as cough, breathlessness, chest pain and exercise intolerance [41,42]. Affected children may manifest recurrent lower respiratory tract infections with resultant worsening damage to their lungs [41]. Lung function testing and chest imaging provide diagnostic insight into the extent and pattern of impairment of lung function and extent of anatomic pathology [46]. Prognosis and natural disease course of PTLD in child and adolescents is not well understood due to lack of structured health systems and longitudinal cohort follow up of affected children after the completion of TB treatment [47].

A recent double-blind, placebo controlled trial was conducted randomising 347 African children with HIV-associated chronic lung disease to one year of weekly azithromycin versus placebo. In the whole cohort azithromycin did not improve lung function, but reduced frequency of all cause acute respiratory exacerbations during one year follow up (hazard ratio 0.50; 95% CI 0.27–0.93; *p* = 0.03) [48]. Notably, 72% of the children in the study had no known history of TB, making it unclear whether these findings are fully generalizable to CALHIV with post-TB chronic lung disease. 

Current public health programs lack structured management protocols for CALHIV who have PTLD as current protocols are those specific to adult chronic obstructive pulmonary disease (COPD); no clear protocols exist specific to children and adolescents with PTLD. There is need for child- and adolescent-focused research to guide optimal interventions and packages for the comprehensive care of CALHIV in various settings who survive TB but experience continuing morbidity and reduced quality of life due to PTLD [43,47].

## 8. Conclusions

Despite significant advances in HIV management, emerging data suggest that CALHIV remain at elevated risk for TB even when successfully managed on ART. Continued research is needed to identify effective screening approaches to increase the early detection of TB in CALHIV, improve the accuracy of TB diagnostics, and increase access to ART compatible child friendly treatment and preventive options. Further, the child TB community must recognize that some CALHIV experience post-TB sequelae and still endure the negative impact of TB long after TB treatment has been completed. There is a lack of clear protocols and structured services for their long-term care and a need for child–adolescent specific research to inform the care of PTLD. Aligned with the sustainable development goals to provide a better and sustainable future for all, protocols and services are needed to optimize TB prevention and treatment, and post-TB care among CALHIV, and enable them to reach their full potential. 

## Figures and Tables

**Table 1:** Summary table of studies from high HIV/TB burden settings evaluating TB incidence and outcomes in children and adolescents living with HIV.

**TB Incidence in CALHIV**					
**Author**	**Setting**	**Study Design**	**Population**	**Sample Size**	**Results**
Frigati, 2021 [3]	South Africa	Prospective cohort study	Perinatally infected adolescents living with HIV and HIV-uninfected adolescents	n = 599, 496 HIV positive	Adolescents with perinatally acquired HIV had a TB incidence of 2.2/100 person years. The IRR attributable to HIV was 7.4 (95% CI 1.01 to 53.6)
Tiruneh, 2020 [4]	Southwest Ethiopia	Retrospective observational study	CALHIV who were ART naïve and experienced	n = 800	The incident rate was 7.7 per 100-years, (95% CI 6.3–9.2) in CALHIV on ART, similar to 8.2 per 100 person years (95% CI 6.8–9.8) in ART naïve CALHIV
Mandalakas, 2020 [6]	Lesotho, Eswatini, Botswana, Uganda, Tanzania	Retrospective observational study	CALHIV who were ART naïve and experienced	n = 1160	The incident rate was 2 per 100-person years, which decreased significantly with increases in ART uptake
Martinez, 2020 [7]	34 countries	Systematic review	Children with recent TB exposure with and without HIV	137,647 TB-exposed children	HIV was associated with an incident aHR of 5.31, 95% CI 2.39–11.81
Nduba, 2020 [5]	Kenya	Prospective observational cohort	Infants with and without HIV	n = 2900	Infants with HIV had an adjusted HR of 4.71, 95% CI 2.13–10.4
**TB Outcomes in CALHIV**					
**Author**	**Setting**	**Study Design**	**Population**	**n**	**Results**
Osman, 2021 [8]	South Africa	Retrospective review of programmatic TB outcomes	Children with TB, with and without HIV	n = 729,463, 102,643 HIV positive	HIV was associated with mortality: aHR = 5.11, 95% confidence interval 4.71–5.55 on ART and 7.99, 95% CI 7.02–9.09 off ART
Onyango, 2018 [15]	Kenya	Retrospective review of programmatic TB outcomes	Children with TB, with and without HIV	n = 24,216, 5991 HIV positive	HIV was associated with an aHR of death 3.69, 95% CI 3.14–4.35 on ART and an aHR of 4.84, 95% confidence interval 3.59–6.91 off ART

Abbreviations: ART: antiretroviral treatment, CALHIV: children and adolescents living with HIV, TB: tuberculosis, IRR: incidence rate ratio, aHR: adjusted hazard ratio.

**Table 2:** Suggested dose adjustment for children requiring rifampicin and antiretroviral therapy (31).

Drug	Induction	Effect	Suggested Adjustment	Comments
**Integrase strand inhibitors**				
Dolutegravir	UGT1A1 CYP3A	Reduced AUC and trough	Twice daily dose	Dose depends on the formulation used
Raltegravir	UGT1A1	Reduced AUC and trough	Doubling each dose	Dose depends on the formulation used
Bictegravir	UGT1A1 CYP3A	Reduced AUC and trough	Avoid co-treatment fairliewangNo mitigating strategies proven to overcome interactions	Do not use
Elvitegravir/cobicistat		No data	No data	Do not use
**Non-nucleoside reverse transcriptase inhibitors**				
Efavirenz	CYP 2B6fairliewangCYP 2A6fairliewangUGT2B7	Reduced AUC and trough	No dose change	INH effects may contract some of the effects of induction
Nevirapine	CYP 3AfairliewangCYP 2B6	Reduced AUC and trough	Increase the dose to 200 mg/m^2^/dose bd	No longer recommended
Doravirine	CYP 3A4	Reduced AUC and trough	No mitigating strategies proven to overcome interactions	Do not use
Etravirine	CYP 3A4	Reduced AUC and trough	No mitigating strategies proven to overcome interactions	Do not use
Rilpivirine	CYP 3A4	Reduced AUC and trough	No mitigating strategies proven to overcome interactions	Do not use
**Protease inhibitors**				
Lopinavir/ritonavir 4:1	CYP 3A	Reduced AUC and trough	Liquid formulation and solid granule formulations fairliewangAdd ritonavir to achieve a 1:1 ratio	Liquid and solid granule or pellet formulations should not be given 3 times a day or at double the dose
			Solid tablet formulations: doubling the dose	Tablets should not be crushed
Atazanavir	CYP 3A4	Reduced AUC and trough	No mitigating strategies proven to overcome interactions	Do not use
Darunavir	CYP 3A4	Reduced AUC and trough	No mitigating strategies proven to overcome interactions	Do not use
**Non-nucleoside reverse transcriptase inhibitors**				
Abacavir	UGT	Reduction	No change in dose	
Zidovudine	UGT	Reduction	No change in dose	
Tenofovir disoproxil fumarate	P-gp	Reduction	No change in dose	
Tenofovir alafenamide	P-gp	Reduction	No change in dose	

CYP: cytochrome; AUC: area under the curve; UGT: UDP-glucuronosyl transferases; P-gp: pglycoprotein.

## Data Availability

Not applicable.
